# Minimal Interspecies Interaction Adjustment (MIIA): Inference of Neighbor-Dependent Interactions in Microbial Communities

**DOI:** 10.3389/fmicb.2019.01264

**Published:** 2019-06-11

**Authors:** Hyun-Seob Song, Joon-Yong Lee, Shin Haruta, William C. Nelson, Dong-Yup Lee, Stephen R. Lindemann, Jim K. Fredrickson, Hans C. Bernstein

**Affiliations:** ^1^Biological Sciences Division, Pacific Northwest National Laboratory, Richland, WA, United States; ^2^Department of Biological Sciences, Tokyo Metropolitan University, Hachioji, Japan; ^3^Bioprocessing Technology Institute, Agency for Science, Technology and Research, Singapore, Singapore; ^4^School of Chemical Engineering, Sungkyunkwan University, Suwon, South Korea; ^5^Whistler Center for Carbohydrate Research, Department of Food Science, Purdue University, West Lafayette, IN, United States; ^6^Department of Nutrition Science, Purdue University, West Lafayette, IN, United States; ^7^Faculty of Biosciences, Fisheries and Economics, UiT – The Arctic University of Norway, Tromsø, Norway; ^8^The Arctic Centre for Sustainable Energy, UiT – The Arctic University of Norway, Tromsø, Norway

**Keywords:** context dependence, network inference, rule-based approach, microbiome modeling, microbial ecology

## Abstract

An intriguing aspect in microbial communities is that pairwise interactions can be influenced by neighboring species. This creates context dependencies for microbial interactions that are based on the functional composition of the community. Context dependent interactions are ecologically important and clearly present in nature, yet firmly established theoretical methods are lacking from many modern computational investigations. Here, we propose a novel network inference method that enables predictions for interspecies interactions affected by shifts in community composition and species populations. Our approach first identifies interspecies interactions in binary communities, which is subsequently used as a basis to infer modulation in more complex multi-species communities based on the assumption that microbes minimize adjustments of pairwise interactions in response to neighbor species. We termed this rule-based inference minimal interspecies interaction adjustment (MIIA). Our critical assessment of MIIA has produced reliable predictions of shifting interspecies interactions that are dependent on the functional role of neighbor organisms. We also show how MIIA has been applied to a microbial community composed of competing soil bacteria to elucidate a new finding that – in many cases – adding fewer competitors could impose more significant impact on binary interactions. The ability to predict membership-dependent community behavior is expected to help deepen our understanding of how microbiomes are organized in nature and how they may be designed and/or controlled in the future.

## Introduction

Microbial communities develop emergent properties through complex networks of interspecies interactions, which cannot be understood by simply examining the behavior of all members individually. Microbial interaction dynamics underpin community functions associated with various critical processes for society, such as environmental sustainability (Gougoulias et al., 2014; Rousk and Bengtson, 2014), human health (Cho and Blaser, 2012; Young, 2017), and bio-manufacturing industry (Gustavsson and Lee, 2016; Schmidt-Dannert and Lopez-Gallego, 2016; Valdivia et al., 2016). Predicting – and ultimately engineering – stable and productive functioning of microbial communities requires a fundamental understanding of how microbes influence each other and self-organize into complex interaction networks in response to environmental changes and compositional shifts (Lindemann et al., 2016; Song, 2018; Song et al., 2018).

Microbe-microbe interactions can be either bidirectional or unidirectional and hence can be systematically classified as pairs: positive-positive (mutualism and synergism), negative-negative (competition), positive-negative (antagonism such as predation and parasitism), positive-neutral (commensalism), negative-neutral (amensalism), and neutral-neutral (no significant interaction). Types and strength of interspecies interactions can be computationally inferred from experimental data with respect to these categories. These data are typically observed as population abundances and growth kinetics, because comprehensive molecular-level multi-omics data that relate to microbial interactions are rare. Despite an increasing number of inference methods reported hitherto (Faust and Raes, 2012; Song et al., 2014; Faust et al., 2015), reliable, comprehensive prediction still faces challenges. Difficulties often arise from insufficient resolution of input data (species population or abundance), typically associated with data sparsity and noise, as well as the uncertainty in differentiating direct ecological interactions from indirect relationships (Li et al., 2016). However, in our view, more fundamental challenges lie in the lack of our understanding of how the microbial interactions in a community are organized as an interaction network and how they affect complex ecological dynamics (Konopka et al., 2015).

Researchers have sought to develop new tools to manage these challenges from both experimental and modeling perspectives. One such tool has been the use of combinatorial co-cultivation, which provides insight into core pairwise interactions between cultivable microbes (Wintermute and Silver, 2010; Bernstein and Carlson, 2012; Grosskopf and Soyer, 2014; Khan et al., 2018). Behavioral changes in single species that are treated with specific binary partnerships can be synergistically examined through experiments (Bernstein et al., 2012, 2017) and modeling analyses (Song et al., 2014; Henry et al., 2016). In many cases, however, knowledge from simple binary cultures is not directly translatable to complex, multi-species communities due to the effect of additional members. That is, interaction coefficients between species identified in simple and complex communities often show quantitative and even qualitative differences, which is defined here as *neighbor dependence of interactions*. In community ecology, this aspect of interactions has been studied as part of a broader concept termed context dependence, which includes the impact of spatial and abiotic gradients, as well as biotic factors (Chamberlain et al., 2014).

A prime example of neighbor-dependent interaction in the murine and human gut microbiome showed that, after antibiotic treatment, a pathogen *Clostridium difficile* in the digestive tract suppressed the growth of residing intestinal bacteria by producing cytotoxin (Buffie et al., 2015). Intestinal bacteria developed resistance to *C. difficile*, however, by the addition of another species, *Clostridium scindens*, which produces secondary bile acids that can impair the growth of the pathogen. Microbial interactions modulated by additional members are often implied by population shifts. In colonic fermentation of carbohydrates not accessible to degradation by human enzymes, population sizes of lactobacilli incapable of directly degrading long-chain inulin were modulated by organisms possessing enzymes to degrade these polysaccharides (Rossi et al., 2005). Furthermore, differential metabolism of members of the altered Schaedler flora, a well-studied model microbiome of mice, was observed when members grown in fresh media vs. spent media from other members of the community (Biggs et al., 2017). These effects are not specific to the gut microbiota; synergistic growth responses were observed for ternary communities of environmental microbes in fermentations of cellulose and xylan (Deng and Wang, 2016).

The above examples indicate that interaction coefficients between two species are not constant, but can vary as a function of community membership and species population density. Changes in pairwise interactions by a third-party organism can be caused in two ways (Wootton, 1993; Momeni et al., 2017): (1) interaction modification and (2) interaction chain. In the first type, a third-party organism affects interaction by forming a multi-way relationship, which is thus known as higher-order interaction (Kato et al., 2008; Voit, 2013; Zelezniak et al., 2015; Bairey et al., 2016; Grilli et al., 2017; Levine et al., 2017). In the second type, a third-party organism does not directly participate in interaction, but affects the density of existing species (thus known as density-mediated indirect interaction). The values of pairwise interaction coefficients in this case can also be modified when the pairwise interaction is driven by a non-linear functional response, while they may remain constant if not. As such, the changes in interactions can be caused by either higher-order interactions or non-linearity. Regardless of what the main source is, understanding such membership-dependent interactions is an ecologically important goal, which requires advanced concepts beyond the scope of traditional theoretical approaches that assume fixed interactions.

The analysis of population data alone does not allow to identify the true source of modification in pairwise interactions. Current practices of ecological community modeling tend to account for this effect by adding higher-order interaction terms. However, that approach substantially increases the number of interaction parameters, making it challenging to reliably infer microbial interactions, particularly for compositionally complex natural communities, which are often composed of hundreds of different taxa or more.

In this article, we propose a novel concept of a rule-based network inference method that enables predicting neighbor-dependent interactions without assuming any functional forms of neighbor dependence of interactions and therefore is scalable to complex communities. Our method predicts the modulation of interaction in complex communities from the knowledge of pairwise interactions derived from binary growth dynamics based upon the assumption that the presence of neighbor species will perturb these intrinsic interactions but only to a minimal degree. We have named this concept the minimal interspecies interaction adjustment (MIIA), the utility of which was demonstrated through comprehensive *in silico* experiments. Specifically, the MIIA hypothesis led to reliable predictions of the change in interaction coefficients in a complex community in an either positive or negative direction. In the analysis of experimental data derived from a microbial community of eight competing soil bacteria (Friedman et al., 2017), we obtained an interesting finding that interspecies relationships can be significantly changed when perturbed by fewer competitors. The modulation of interspecies interactions diminished as competitive neighbors increase. This is important knowledge for the effective control and design of microbial communities. The unique concept of MIIA may contribute to revealing many other intriguing aspects of interspecies interactions in ecological communities beyond microbial systems.

## Materials and Methods

The MIIA-based network inference is implemented following the two steps: (1) the identification of core pairwise interactions in binary cultures, (2) the projection of pairwise interactions identified from binary cultures to complex communities.

### Quantification of Core Pairwise Interactions

We define the interaction coefficient in binary communities ($a_{i,j}^{B}$) as the effect of the presence of species *j* on the growth of species *i*. The relationship between $a_{i,j}^{B}$ and populations can be derived by analyzing the steady state Lotka-Volterra model for single species and a binary community (Supplementary Text), i.e.,

(1) $$\frac{x_{i}^{B}-x_{i}^{A}}{x_{i}^{A}}={\hat{a}}_{i,j}^{B}x_{j}^{B}$$

The left-hand side of the above equation was described as *per capita interaction strength* in the literature (Paine, 1992). Eq. (1) can be rewritten in the following form,

(2) $${\hat{a}}_{i,j}^{B}=\frac{\left(x_{i}^{B}-x_{i}^{A}\right)}{x_{i}^{A}x_{j}^{B}}$$

where $x_{i}^{A}$ is the population of species *i* in the axenic culture (as denoted by the superscript A), $x_{i}^{B}$ and $x_{j}^{B}$ are the populations of species *i* and *j* in the binary culture (as denoted by the superscript B), and ${\hat{a}}_{i,j}^{B}$ denotes the estimated value of $a_{i,j}^{B}$. In the case that species *i* cannot grow axenically (i.e., $x_{i}^{A}$ = 0) or does not allow species *j* to co-grow (i.e., $x_{j}^{B}$ = 0), the effect of species *j* on *i* is not fully identifiable (because the denominator of the right-hand side of Eq. (2) is zero). Throughout this work, we assume that species can grow both alone and together with a partner (so that $a_{i,j}^{B}$ is identifiable).

### Inference of Interactions in Complex Communities

**FIGURE 1:**
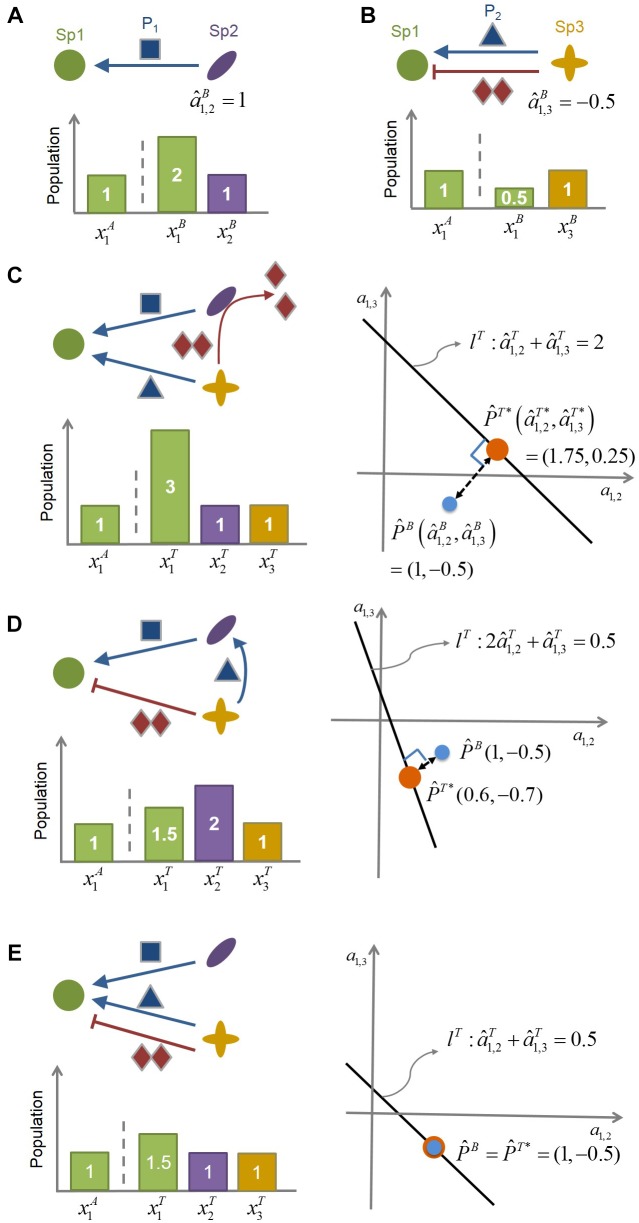
The conceptual illustration of the MIIA concept. **(A)** Species 2 (Sp2) facilitates the growth of Species 1 (Sp1) by providing the promoting compound P1, as can be inferred from the increased population of Sp1 in the presence of Sp2. The interaction coefficient of Sp2 on Sp1 in the binary culture (${\hat{a}}_{1,2}^{B}$) is identified accordingly as being positive. **(B)** Species 3 (Sp3) provides Sp1 with both promoting (P2) and inhibiting compound (I) (such as antibiotics). In this case, the net effect of Sp3 on Sp1 (${\hat{a}}_{1,3}^{B}$) is determined to be negative from the decreased population of Sp1 in the presence of Sp3. **(C–E)** provide three different scenarios of modulation of ${\hat{a}}_{1,3}^{B}$: **(C)** from negative to positive, **(D)** from negative to even more negative, and **(E)** no changes. Linear equation *l*^T^ represents the relationship among interaction coefficients in the ternary community. As represented graphically, the minimal adjustment hypothesis chooses (${\hat{a}}_{1,2}^{T^{*}}$,${\hat{a}}_{1,3}^{T^{*}}$), i.e., the closest point from (${\hat{a}}_{1,2}^{B}$,${\hat{a}}_{1,3}^{B}$), as the estimate of the interaction coefficients in the ternary culture.

For a complex community, a linear interaction model for species *i* can be developed by extending Eq. (1) as follows:

(3) $$\frac{x_{i}^{C}-x_{i}^{A}}{x_{i}^{A}}=\sum_{j=1,\neq i}^{N}a_{i,j}^{C}x_{j}^{C}$$

where the superscript C denotes complex community and *N* is the number of species. Note that when *N* = 2, this equation reduces to Eq. (1). When *N* > 2, no unique determination of $a_{i,j}^{C}$’s is possible for this underdetermined system where the number of unknowns is more than the number of equations. The solution sets satisfying the above equation form an (*N-2*) dimensional hyperplane in the (*N-1*) dimensional space of interaction coefficients, $a_{i,j}^{C}$’s (*j* = 1, …, *N*, ≠ *i*). Based on the minimal adjustment hypothesis, MIIA infers the interspecies interaction coefficients in the complex community (${\hat{a}}_{i,j}^{C}$) as the point on the hyperplane that is closest to the binary interaction coefficients (i.e., ${\hat{a}}_{i,j}^{B}$). We then obtain ${\hat{a}}_{i,j}^{C}$’s as the intersection of Eq. (3) and the orthogonal line passing through the binary point (for illustration, see Figure 1).

### Performance Measures

We used several performance measures for evaluating predictions. Quantitative accuracy of predicted interaction coefficients against true values (if known) was quantified based on cosine similarity. This metric judges the degree of alignment of two vectors, regardless of magnitudes, which is relevant in our case where we focus on the closeness of interaction coefficients between binary and complex communities, instead of aiming at predicting the absolute values. Cosine similarity of two vectors (*a* and *b*), *S*_C_ (*a,b*), can be calculated as follows:

(4) $$S_{C}(a,b)=\frac{a\cdot b}{{\left\|a\right\|}_{2}{\left\|b\right\|}_{2}}$$

where the dot (⋅) operator denotes the vector inner product, and || ||_2_ represents the *l*2- (or Euclidean) norm. As a related measure, the difference of interaction coefficient vectors (*a* and *b*) was quantified by cosine distance, *D*_C_(*a,b*), which is defined as follows:

(5) $$D_{C}(a,b)=1-S_{C}(a,b)$$

As an additional metric for performance evaluation, we used the prediction accuracy for the direction of interaction modulation as defined below:

(6) Accuracy =TD/(TD+FD)

where TD and FD denote the numbers of correct and incorrect predictions of the direction of interaction modulation. We define the direction of interaction modulation as the sign of the following quantities, i.e.,

(7) $$\begin{array}{l} m_{i,j}\equiv a_{i,j}^{C}-a_{i,j}^{B}\\ {\hat{m}}_{i,j}\equiv {\hat{a}}_{i,j}^{C}-{\hat{a}}_{i,j}^{B} \end{array}$$

where *m*_i,j_ denotes the modulation level of interaction between species *i* and *j*, and ${\hat{m}}_{i,\;j}$ is the estimate of *m*_i,j_. The positive *m*_i,j_ implies that the effect of species *j* on species *i* becomes more cooperative or less inhibitive in the complex community, relative to in the binary community. Similarly, the negative *m*_i,j_ implies that the effect of species *j* on species *i* becomes less cooperative or more inhibitive in the complex community, relative to in the binary community.

### Generation of *in silico* Population Data

For testing the accuracy of MIIA, we used simulated data generated using a generalized Lotka-Volterra (gLV) model given in the following form:

(8) $$\frac{dx_{i}}{dt}=x_{i}r_{i}\left(1+\sum_{j=1}^{N}a_{i,j}x_{j}\right),\;i=1,2,\cdots ,N$$

where *x*_i_ denotes the population of species *i, r*_i_ is the growth rate in the absence of other species, and *a*_i,j_ represents the influence of species *j* on the growth of species *i*. In all simulations, we fixed the growth rates with a non-zero constant (i.e., *r*_1_ = … = *r*_n_ = *r* > 0). We also fixed the diagonal terms of interaction matrices to be negative (i.e., *a*_1,1_ = … = *a*_n,n_ = -*d* < 0) so that the community is stabilized due to the dominance of intraspecific competition (May, 1972). Pairwise interaction coefficients in binary combinations ($a_{i,j}^{B}$) were assigned with random variables following the normal distribution, *N* (0, α). To account for neighbor-dependent interactions, we assigned new random values taken from *N* ($a_{i,j}^{B}$, β) to the interaction coefficients in complex communities ($a_{i,j}^{C}$). As standard conditions, we set *r* = 1, α = 0.1, and varied β from 0 to 1. We set *d* = $\sqrt{N}$ based on theoretical stability criteria for complex communities (May, 1972; Allesina and Tang, 2012). For each parameter set, we generated 30 independent sets of interaction coefficients for binary ($a_{i,j}^{B}$) and complex communities ($a_{i,j}^{C}$). For each case, population data were obtained by solving Eq. (8) over a given time span to ensure that all species reach the steady state. Population data at this end point were used as input for MIIA. We discarded cases where any of the member species goes extinction.

### Null Model-Based Prediction

We considered null models as a basis for comparatively evaluating the performance of our prediction. Similar to the method describe above, we designed null models for complex communities by assigning random numbers (following a normal distribution centered at $a_{i,j}^{B}$) to interaction coefficients. The performance of null models and MIIA prediction was evaluated by comparing their interaction coefficients with previously determined true values (as described in the foregoing section).

### Experimentally Synthesized Eight-Member Community

To evaluate MIIA against experimental data, we took advantage of combinatorial co-culture data collected from the bacterial community presented by Friedman et al. (2017). The member species include eight soil bacteria: *Enterobacter aerogenes* (Ea), *Pseudomonas aurantiaca* (Pa), *Pseudomonas chlororaphis* (Pch), *Pseudomonas citronellolis* (Pci), *Pseudomonas fluorescens* (Pf), *Pseudomonas putida* (Pp), *Pseudomonas veronii* (Pv), and *Serratia marcescens* (Sm).

## Results

### Prediction of Dramatic Changes of Interspecies Interactions Depending on the Role of Third-Party Species

**FIGURE 2:**
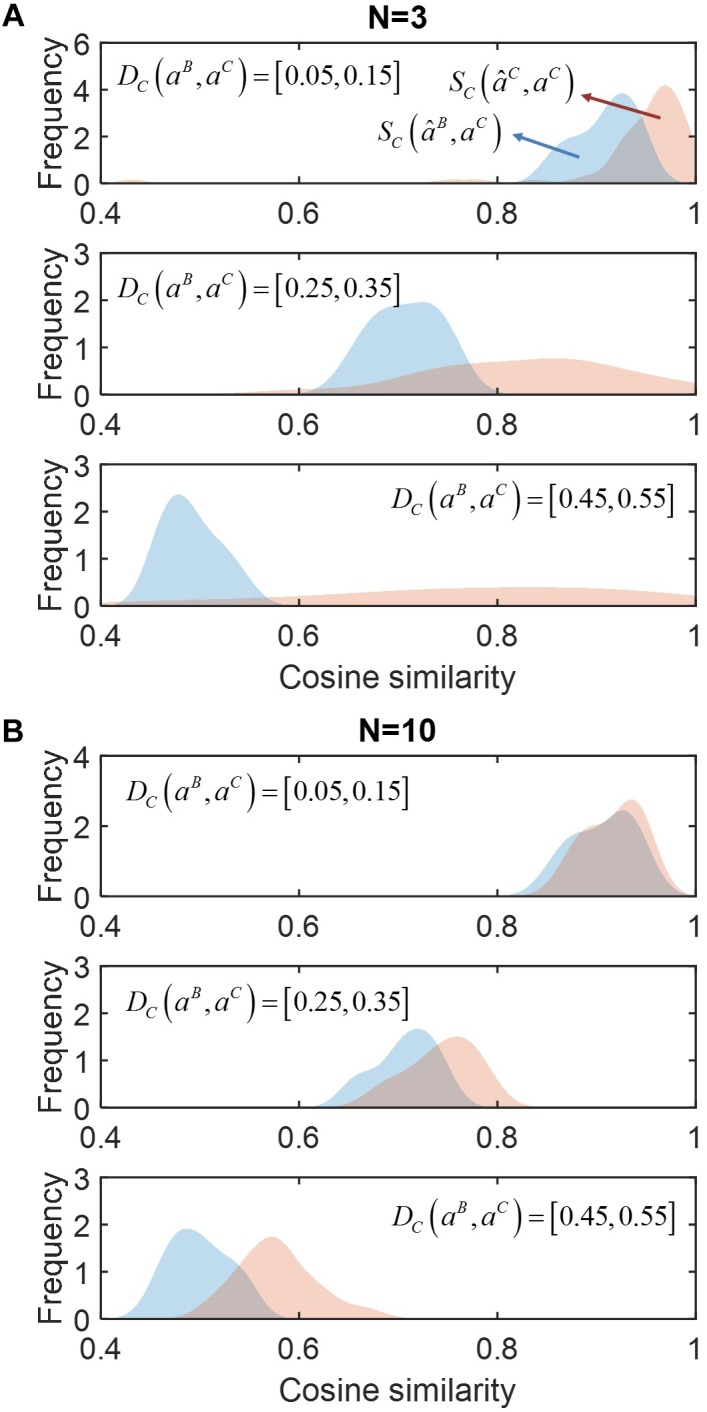
The effect of accounting for member dependence of interactions on predictions for complex communities of different sizes (*N*). **(A)** *N* = 3, and **(B)** *N* = 10. For each case, simulated data were sampled into three groups depending on the level of interaction modulation from binary to complex communities as quantified by cosine distance *D*_C_ (*a*^B^, *a*^C^), i.e., slight (top), moderate (middle) and significant (bottom) modulations. The performance of MIIA prediction (*â*^C^) and member dependence-neglected estimation (*â*^B^) can be evaluated by cosine similarities between their predictions with true interaction coefficients, i.e., *S*_C_ (*â*^C^, *a*^C^) and *S*_C_ (*â*^B^, *a*^C^), the distributions of which were displayed in red and blue, respectively, and obtained by non-parametric kernel-smoothing method.

To illustrate the role that the MIIA concept can play in predicting the effect of population shifts on microbial interactions, we considered a hypothetical community composed of three-member species (Sp1, Sp2, and Sp3) (Figure 1) and assumed the following *binary* interaction scenarios: (1) Sp2 exerts on Sp1 is positive (Figure 1A); (2) Sp3 provides two counter-affecting compounds that exert positive and negative effects, respectively, on Sp1, with the net effect being negative (Figure 1B). We used this virtual community to examine how the effect of Sp3 on Sp1 can be changed in a ternary culture depending on the role of Sp2 as considered in the following three scenarios:

Case 1: Negative to positive. Sp2 degrades the Sp3-derived compound that inhibits the growth of Sp1 (Figure 1C).

Case 2: Negative to even more negative. Sp2 exploits the Sp3-derived compound that promotes the growth of Sp1 (Figure 1D).

Case 3: No change. Sp2 does not interfere with Sp3 (Figure 1E).

In the situation where the mechanistic understanding of the role of Sp2 assumed above is lacking, the question is how to quantitatively identify these modulations from the population data. As described in Methods, the MIIA predicts the effect of neighbors on pairwise interactions through the following two steps. First, we estimate interspecies interactions in binary cultures: the effect of Sp2 on Sp1 in the Sp1-Sp2 binary culture ($a_{1,2}^{B}$) and the effect of Sp3 on Sp1 in the Sp1-Sp3 binary culture ($a_{1,3}^{B}$), i.e.,

(9) $$\begin{array}{l} x_{1}^{B}=x_{1}^{A}+{\hat{a}}_{1,2}^{B}x_{2}^{B}\\ x_{1}^{B}=x_{1}^{A}+{\hat{a}}_{1,3}^{B}x_{3}^{B} \end{array}$$

From the binary population data in Figure 1A,B (see the bar charts), Eq. (9) yields ${\hat{a}}_{1,2}^{B}$ = +1 and ${\hat{a}}_{1,3}^{B}$ = -0.5. Second, we extend Eq. (9) to the three-member community as follows:

(10) $$x_{1}^{T}=x_{1}^{A}+{\hat{a}}_{1,2}^{T}x_{2}^{T}+{\hat{a}}_{1,3}^{T}x_{3}^{T}$$

where the superscript T denotes the ternary culture. That is, ${\hat{a}}_{1,2}^{T}$ (or ${\hat{a}}_{1,3}^{T}$) represents the estimated effect of Sp2 (or Sp3) on Sp1 in the presence of Sp3 (or Sp2). For the three scenarios considered above, Eq. (10), respectively, becomes

(11) $$\begin{array}{l} \mathrm{Case 1}:\;{\hat{a}}_{1,2}^{T}+{\hat{a}}_{1,3}^{T}=2\\ \mathrm{Case 2}:\;2{\hat{a}}_{1,2}^{T}+{\hat{a}}_{1,3}^{T}=0.5\\ \mathrm{Case 3}:\;{\hat{a}}_{1,2}^{T}+{\hat{a}}_{1,3}^{T}=0.5 \end{array}$$

All cases in Eq. (11) are underdetermined in that the number of unknowns is greater than the number of equations, for which solution sets are infinite, forming straight lines (*l*^T^) in the space of interaction coefficients *a*_1,2_ and *a*_1,3_. For each case, the minimal adjustment hypothesis infers interaction coefficients in the ternary culture as the point on *l*^T^, which is geometrically closest to $\left({\hat{a}}_{1,2}^{B},{\hat{a}}_{1,3}^{B}\right)$. That is, we obtain $\left({\hat{a}}_{1,2}^{T},{\hat{a}}_{1,3}^{T}\right)$ through the orthogonal projection of $\left({\hat{a}}_{1,2}^{B},{\hat{a}}_{1,3}^{B}\right)$ onto *l*^T^. The resulting value of $\left({\hat{a}}_{1,2}^{T},{\hat{a}}_{1,3}^{T}\right)$ was (1.75, 0.25) and (0.6, −0.7) for Cases 1 and 2, respectively. These results show that the MIIA concept can correctly lead to the prediction of the assumed change in the effect of Sp3 on Sp1 from binary to ternary cultures, i.e., “negative to positive” (Case 1) or “negative to even more negative” (Case 2). The effect of Sp2 on Sp1 was also predicted to change in the ternary culture but only as to the magnitudes, not the sign. For Case 3, where no modulation of interactions occurs between binary and ternary cultures, the point of $\left({\hat{a}}_{1,2}^{T},{\hat{a}}_{1,3}^{T}\right)$ was estimated the same as $\left({\hat{a}}_{1,2}^{B},{\hat{a}}_{1,3}^{B}\right)$.

Graphical illustration of interspecies interactions in Figure 1C to 1e suggests that the degree of modulation can be significant or negligible depending on the relative strength of binary interactions among residing species vs. interactions between residing species and new members. It also implies that stronger binary interactions may be likely to be more conserved in complex communities than weaker interactions, while not always.

### Robust Prediction of the Direction of Interaction Modulation

To understand how the MIIA concept is applied to complex communities in a more general context, we generated a larger suite of virtual community data using a gLV model (Song et al., 2014). This data serves as a useful resource for benchmarking the performance and predictive power of our new inference concept because a “ground truth” is known *a priori* (Lingeman and Shasha, 2012).

We found that the interaction coefficients estimated for binary communities using our method are accurate in all cases considered in this work, as judged by the unity value of cosine similarity between *â*^B^ and *a*^B^, *S*_C_(*â*^B^,*a*^B^). This indicates that the accuracy of our prediction in this work is strongly dependent on how *â*^B^ is translated into *â*^C^ (i.e., the validity of the minimal adjustment hypothesis).

In Figure 2, we compared *S*_C_(*â*^B^,*a*^C^) (red) and *S*_C_(*â*^C^,*a*^C^) (blue) for communities composed from different numbers of species (*N*): (a) *N* = 3, (b) 10 (see Supplementary Figure S1 for the cases of *N* = 4 and 5). Regardless of the number of species, *S*_C_ (*â*^C^,*a*^C^) was consistently shown higher than *S*_C_ (*â*^B^,*a*^C^), indicating that accounting for neighbor dependence based on the minimal adjustment hypothesis was crucial for improving prediction. Such improvement was more significant when community size was smaller (i.e., *N* = 3 or 4). Interestingly, in this case, *S*_C_ (*â*^C^,*a*^C^) showed wider variation when the level of membership-dependent change was significant, while no such variation was observed when *N* was large. In Supplementary Figure S2, we provided simple examples of the MIIA prediction of full interaction matrices in binary and ternary communities along with the cosine similarity.

**FIGURE 3:**
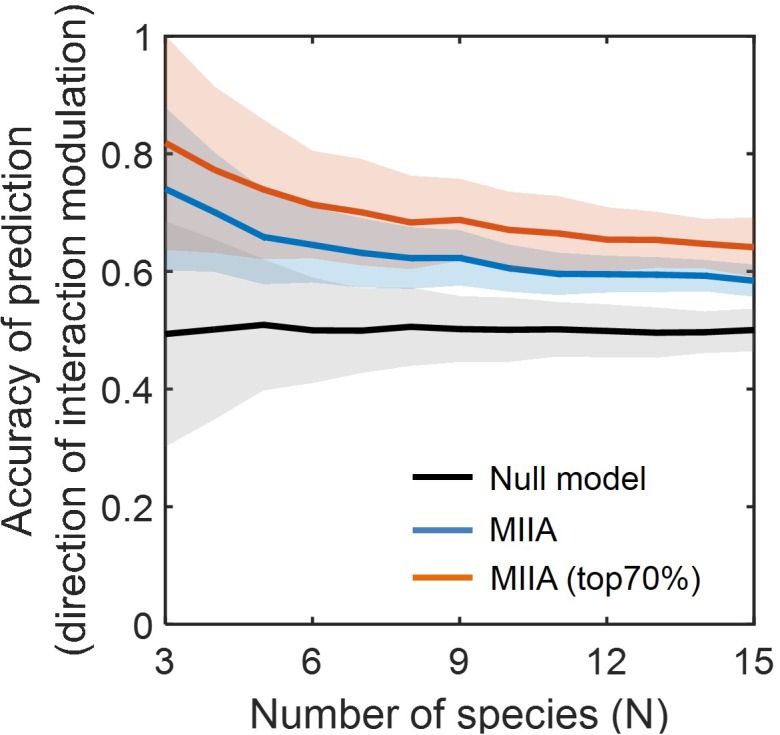
Accuracy of the predicted direction of interaction modulation. For each community of a given size (varying from 3 to 15), simulated data were sampled with the level of modulation between binary and complex communities quantified by *D*_C_ (*a*^B^, *a*^C^) varied from 0.05 to 0.55. Lines and colored shades denote average performances and standard deviations. Black line denotes the prediction by null model, and blue and red lines are MIIA-based predictions for the entire set of interaction coefficients (blue) and for a subset that contains only top 70% modulation (i.e., selected based on the 30% cut-off criterion of the largest modulation) from binary to complex communities.

Figure 3 shows the prediction accuracy of the MIIA-based inference with respect to direction of interaction modulation, which was significantly higher in comparison to the null model based on the simple random sampling (see section “Materials and Methods”). The accuracy of MIIA varied from 0.75 to 0.6 as the community size (*N*) increased from 3 to 15. Interestingly, the accuracy was significantly increased (i.e., to the range of 0.7 to 0.8) when the evaluation was confined to a subset of predictions that show appreciably large modulation (e.g., top 70%). In all cases, the performance of prediction exhibited a decreasing trend as the number of species increases, the rate of decrease was diminished, however, after a certain number of species (i.e., *N* = 5).

As a special case of the modulation of interaction, one would be interested in predicting the sign change in interaction coefficients. While the fundamental change of interspecies relationship from positive to negative or the converse is an interesting ecological event (as exemplified in Figure 1), the sign change *per se* may not be an appropriate measure unless the level of modulation is significant (Supplementary Figures S3, S4).

**FIGURE 4:**
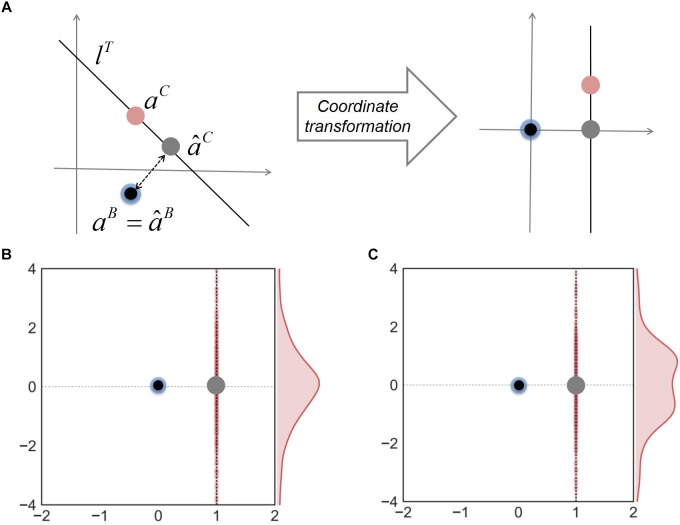
The relationship among interaction coefficients for binary and complex communities. **(A)** Relocation of interaction coefficients through coordinate transformation from the original space (left) to the new space (right). Blue and red dots denote true interaction coefficients for binary and complex communities; black and gray dots are predicted interaction coefficients for binary and complex communities. Two bottom panels show the results of coordinate transformation for three-member communities, interaction coefficients of which were generated by random numbers following **(B)** normal and **(C)** uniform distributions, respectively.

Finally, to address how the minimal adjustment hypothesis works to improve predictions of neighbor-dependent interaction modulation, we examined the relationship among the predicted and true interaction coefficients for binary and complex communities. In order to visualize data effectively, we made coordinate transformation to project all interaction coefficients onto the same space where *â*^B^ and *a*^B^ are located to be the origin and *l*^T^ is a vertical line along the y-axis (Figure 4A). Figure 4B shows that in these new coordinates, *a*^C^ is normally distributed along a vertical direction; consequently, the line from *â*^B^ to *â*^C^ (gray dot) is orthogonal to the distribution of *a*^C^. A similar result was obtained when we generated interaction coefficients in complex communities using random numbers following a “uniform” distribution (Figure 4C) (instead of normal), implying that a good approximation by orthogonal projection was not caused by the way interaction coefficients were generated. These results together show that the MIIA provides a fairly robust predictions for both *a*^B^ and *a*^C^, but also highlights the orthogonal projection of *â*^B^ onto *l*^T^ (i.e., the minimal adjustment hypothesis) offers the best result on average.

### Effect of Measurement Noise

In general, population data contains measurement noise, which often negatively affects the performance of network inference algorithms. To identify the degree at which MIIA prediction deteriorate by measurement error, we generated new datasets by accounting for multiplicative lognormal noise as follows:

(12) $$x_{\mathrm{noise}}=\{\begin{array}{l} x\exp\left[N\left(0,\delta_{\mathrm{noise}}^{A,B}\right)\right]\mathrm{for populations in axenic and binary cultures}\\ x\exp\left[N\left(0,\delta_{\mathrm{noise}}^{C}\right)\right]\mathrm{for populations in complex communities} \end{array}$$

where $\delta_{\mathrm{noise}}^{A,B}$ and $\delta_{\mathrm{noise}}^{C}$ denotes metrics for the level of introduced noise in axenic/binary and complex cultures, respectively. We re-evaluated the MIIA performance with noisy data based on cosine similarity by taking the case of *N* = 5 and β = 0.1, as an example (Supplementary Figure S5). For $\delta_{\mathrm{noise}}^{A,B}$ ≤ 0.01, the accuracy of MIIA prediction was quite robust up to $\delta_{C}^{\mathrm{noise}}$ = 0.1 and linearly decreased afterward (Supplementary Figures S5a–c). For larger values of $\delta_{A,B}^{\mathrm{noise}}$ (≥ 0.1), however, predictions became less accurate (Supplementary Figure S5d). Overall, this result indicates that with relatively more reliable measurements in axenic and binary cultures, the robustness of MIIA prediction can be reasonably high to the noise in complex community data.

### Partnership-Specific Interaction Modulation: Is There Any Overall Trend?

**FIGURE 5:**
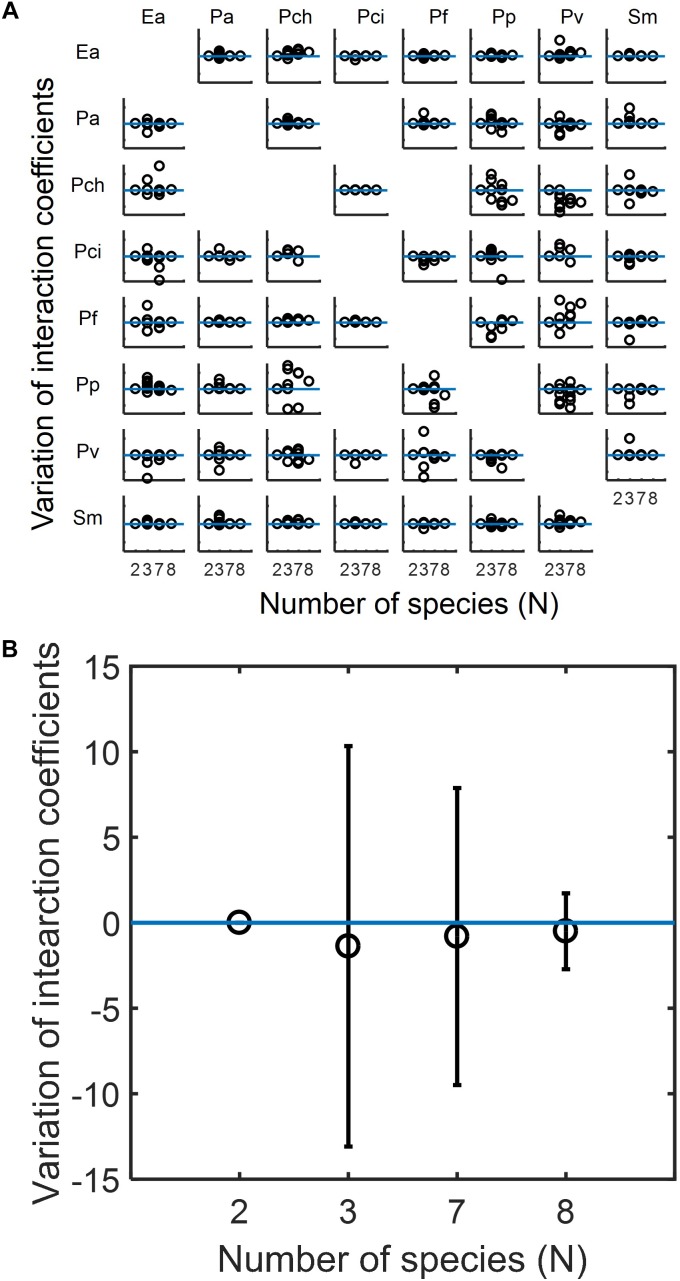
Predicted modulations of interaction coefficients from binary to complex communities composed of competing bacteria considered by Friedman et al. (2017). **(A)** The complete array of the effect of species in columns on species in rows among eight species in binary (*N* = 2), ternary (*N* = 3), septenary (*N* = 7), and octonary cultures (*N* = 8). In each case, interaction coefficients were normalized with respect to the binary coefficients and adjusted to locate binary interaction coefficients to be zero (as denoted by the horizontal blue line) so that *y*-axis implies the deviation of interaction coefficients from core pairwise interactions. Missing off-diagonal subplots represent the cases where binary interactions cannot be identified (see section “Materials and Methods”). **(B)** Overall variation of interaction coefficients in binary and complex communities. All individual plots in **(A)** are merged into one place where open circles and error bars, respectively, denote averages and standard deviations.

Based upon the capabilities of MIIA concept shown in the previous sections, we analyzed experimental data in the literature to understand how the modulation of interaction occurs in microbial communities, the membership of which is subject to variation. We used datasets for eight-member soil bacterial communities studied by Friedman et al. (2017). The MIIA led to the prediction of various patterns of interaction modulation from binary to complex communities as a function of the number of interacting species. As an example, the effect of *Pseudomonas aurantiaca* (Pa) on *Enterobacter aerogenes* (Ea) was predicted to be almost the same in the binary, septenary and octonary cultures, but showed variation in ternary cultures depending upon the identity of the third partner (see also Supplementary Figure S6a). A fundamentally different trend was derived for other combinations. For instance, the effect of *Pseudomonas veronii* (Pv) on Ea in the binary culture was modulated gradually in a positive direction as the number of interacting species increases and showed significant variations in both ternary and septenary cultures (see also Supplementary Figure S6b). A complete array made for all possible combinations confirmed that the modulation of interaction modulation was indeed partnership-specific (Figure 5A).

While diverse patterns were shown individually, we found an interesting trend in the change of interaction modulation when we put all predicted coefficients in one place (Figure 5B). We observed that interaction coefficients varied most significantly in the ternary communities, but the level of variation decreased in the septenary and octonary cultures. That is, the relationship between two competing species in binary cultures are most significantly perturbed when a small number of competitors are introduced, but the level of perturbation progressively decreases as the number of species increases.

As potentially plausible explanations on this counter intuitive finding, pairwise interactions may be less affected when invaded with more competing species because (1) the effect of competition may distribute among all species (and therefore the disruption of particular pairwise interactions can be weaker when species are many), (2) the invaders may not strongly compete with residing species due to the increased level of competition among themselves. Interestingly, this trend of interaction modulation showed some relationship with phylogenetic distance between the effector and added species: the distribution of phylogenetic distances was shown broader in smaller communities, while the same was relatively narrower in large communities, due to the averaging effect among added species (Supplementary Figure S7). It is an interesting future work to examine whether our finding obtained from this particular case is also observable in other communities.

## Discussion

We present and critically evaluate MIIA a new concept of network inference that addresses the following, related ecological questions: (1) how are interspecies interactions modulated by the shifts in community composition and species populations? and (2) to what extent can interspecies relationships observed in simple cultures be translated into complex communities? Our results show that pairwise interactions in binary communities can serve as meaningful references for predicting interactions in more complex communities, e.g., when translated through the minimal adjustment assumption. It needs to be noted that the term “minimal” does not imply little changes because as shown in our examples, this minimal assumption enables predicting dramatic changes of interactions, encouragingly from the analysis of population changes in communities, even without fundamental knowledge of underlying interaction mechanisms.

Beyond a conceptual basis for understanding context-dependent microbial interactions, our work also provides critical information practically useful for the control and design of microbial interactions and community function. First, we demonstrated that our framework can provide reliable prediction of the direction of interaction modulation, i.e., how pairwise interactions are weakened or strengthened under the influence of third-party organisms. Second, through the case study of competing bacteria, we also found that the competitive relationship can be significantly modulated when perturbed by a small number of species, but the level of modulation diminishes as the number of new competing members increases. These results together can serve as useful lessons for a rational engineering of microbial community functioning through compositional and population changes.

How can the minimal adjustment be rationalized from an ecological perspective? While this is an open question, we conjecture that the answer would be associated with stability characteristics and energetics of microbial interaction networks. MIIA implicitly assumes that pairwise interactions observed from a given binary environment are unique and stable – otherwise, taking them as references may not be reasonable. When perturbed by a new member, the community’s stability landscape will be altered and the previous interaction network may be unstable or less stable, leading to the reorganization of interaction network toward a more stable state. In this reorganization, species may need to change their enzyme settings to adapt to a new environment (created by new members) at the expense of energy. The solution that MIIA chose, minimizes the adjustment in pairwise interactions, implying that network reorganization will occur toward spending minimal energy, the amount of which is assumed to be proportional to the level of modulation. In a different area, the minimal adjustment concept has been employed for predicting the metabolic response of individual microorganisms to gene knockouts (Segre et al., 2002; Shlomi et al., 2005). However, our approach is fundamentally different in that we apply this idea to microbes that (can) change their function based on the context, whereas genes generally do not.

Higher-order interactions are a major factor that exerts a profound impact on pairwise interactions. Ideally, evaluation of this effect would require the use of simulated data generated from properly formulated higher-order interaction models. However, identification of appropriate values of interaction parameters for ensuring stable population profiles was a taxing task, particularly for complex communities composed of a large number of member species. In this work, we instead used random variations of interaction parameters to realize neighbor-dependent interactions, including the cases where modulated interaction coefficients show significant deviations from binary parameters (as controlled by the parameter β up to 1), which therefore provided unbiased datasets for assessing the validity of our method. The resulting datasets generated as such could be broadly useful because our focus is not necessarily limited to higher-order interactions but includes non-linear interactions.

The understanding of context dependence of interactions is a key component for making microbial ecology as a more predictive discipline. Toward this end, the generalizability of the proposed concept will be tested through further case studies in diverse contexts and applications including natural microbial communities (such as in human body, soil, and marine environment), as well as engineered synthetic consortia. Modern meta-omics analyses and other advanced experimental techniques offer powerful tools for quantifying interspecies interactions in microbial communities, the resulting data of which will serve as a valuable basis for informing and validating computational network inference approaches such as the one we proposed in this work.

## Author Contributions

H-SS and SH designed the research. H-SS developed the MIIA concept and performed the simulations. J-YL made a critical contribution to the analysis of results. WN, J-YL, and SL contributed to the phylogenetic analysis of soil organisms. H-SS drafted out the manuscript, which was edited by HB, SL, JF, SH, and D-YL. All authors read and approved the final manuscript.

## Conflict of Interest Statement

The authors declare that the research was conducted in the absence of any commercial or financial relationships that could be construed as a potential conflict of interest.
