# Supplementary material for: Minimal Interspecies Interaction Adjustment (MIIA): Inference of Neighbor-Dependent Interactions in Microbial Communities
Source: Front Microbiol. 2019 Jun 11;10:1264. doi: 10.3389/fmicb.2019.01264 (PMC6584816; doi:10.3389/fmicb.2019.01264)
Supplement: Supplementary file 1 [file Data_Sheet_1.docx]

**Supplementary Text**

**Formula for Estimating Binary Interaction Coefficients**

A generalized Lotka-Volterra model for a community composed of *n* species can be written as follows:

$$\frac{dx_{i}}{dt}=x_{i}r_{i}\left(1+\sum_{j} a_{i,j}x_{j}\right), i=1,...,n$$

In steady state (for species *i*),

$$x_{i}r_{i}\left(1+\sum_{j} a_{i,j}x_{j}\right)=0$$

In the cases of the axenic (species *i*) and binary (species *i* and *j*) cultures,

$$x_{i}^{A}r_{i}\left(1+a_{i,i}x_{i}^{A}\right)=0$$

and

$$x_{i}^{B}r_{i}\left(1+a_{i,i}x_{i}^{B}+a_{i,j}^{B}x_{j}^{B}\right)=0$$

where $a_{i,i}$ $\left(<0\right)$ denotes intra-specific interaction coefficient.

Under the conditions where all species can grow both individually and together (i.e., $x_{i}^{A},x_{i}^{B},x_{j}^{B},r_{i}>0$) , the above two equations are respectively reduced to

$$1+a_{i,i}x_{i}^{A}=0$$

and

$$1+a_{i,i}x_{i}^{B}+a_{i,j}^{B}x_{j}^{B}=0$$

Subtraction of Eq. from Eq. leads to

$$a_{i,i}\left(x_{i}^{B}-x_{i}^{A}\right)+a_{i,j}^{B}x_{j}^{B}=0$$

Then,

$$a_{i,j}^{B}=-a_{i,i}\frac{\left(x_{i}^{B}-x_{i}^{A}\right)}{x_{j}^{B}}$$

From Eq. ,

$$a_{i,i}=-\frac{1}{x_{i}^{A}}$$

Finally,

$$a_{i,j}^{B}=\frac{\left(x_{i}^{B}-x_{i}^{A}\right)}{x_{i}^{A}x_{j}^{B}}$$

**Supplementary Figures**


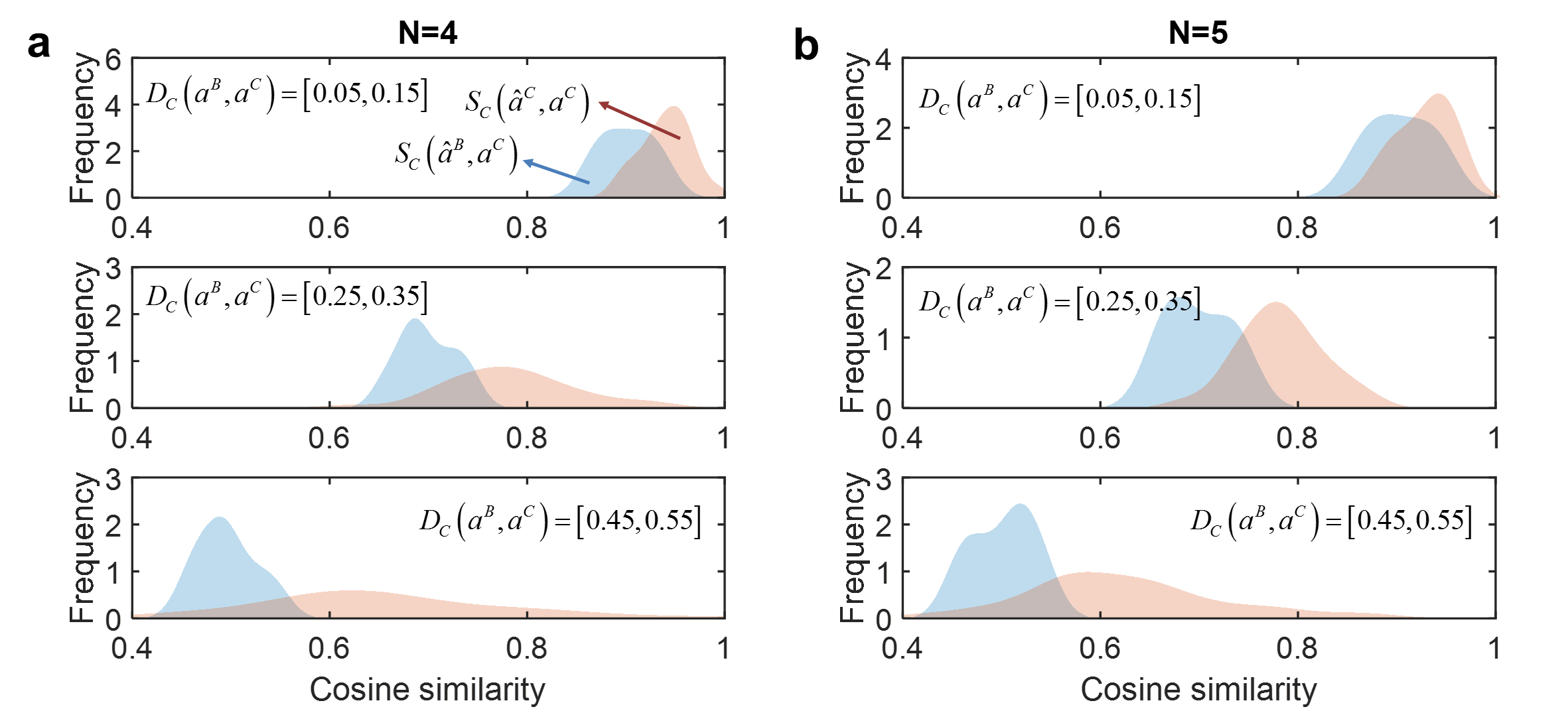


**Figure S1. The effect of accounting for member dependence of interactions on predictions for complex communities of different sizes (*N*).** (a) *N* = 4, and (b) *N* = 5. For all other details, see the caption in Figure 2.


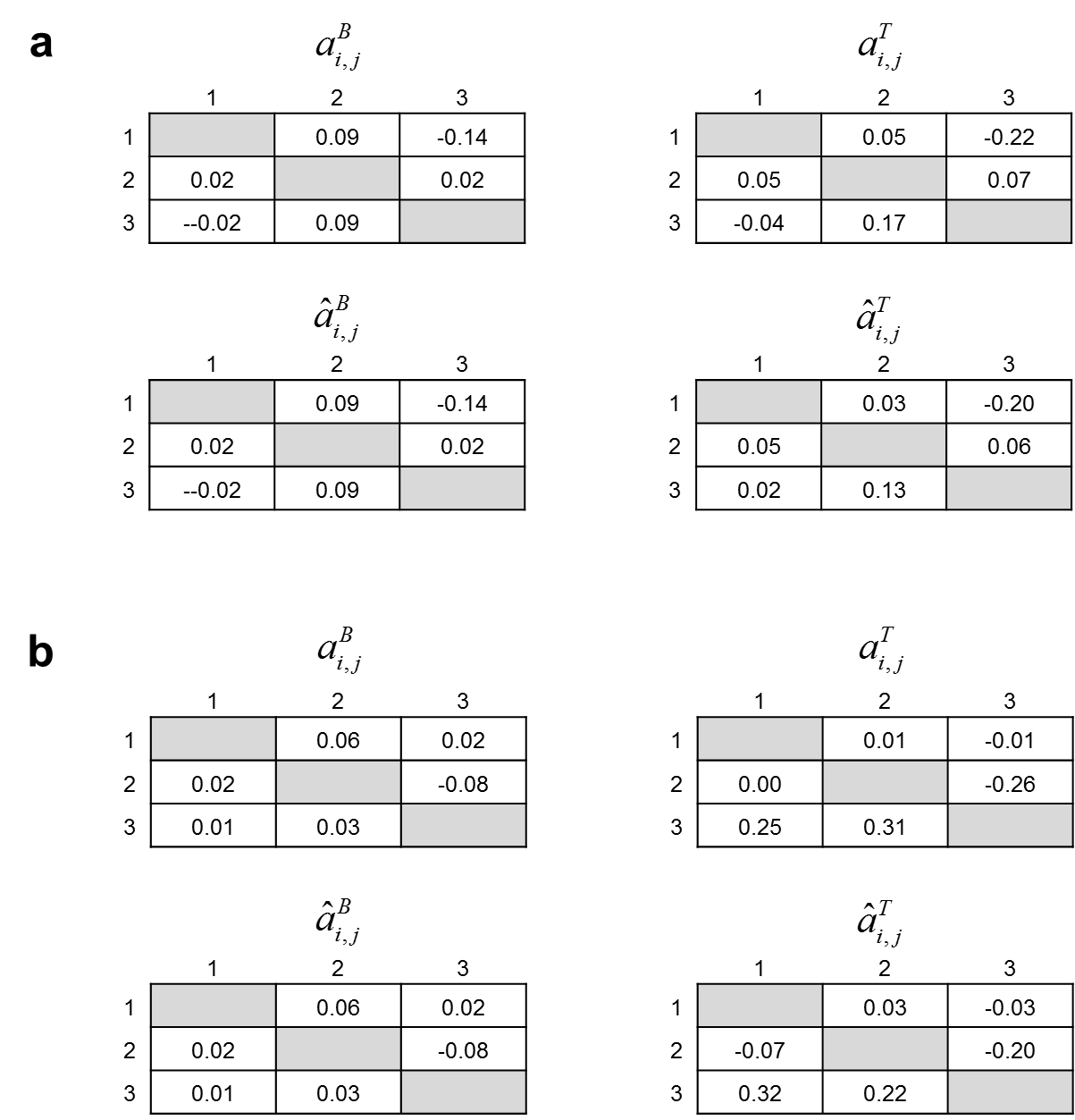


**Figure S2. MIIA predictions of full interaction coefficient matrices for binary and ternary communities.** True interaction coefficient matrices for binary ($a_{i,j}^{B}$) and ternary ($a_{i,j}^{T}$) communities in (a) and (b) were chosen from *in silico* samples generated with $\beta=0.1$ and 0.2 respectively, as described in Methods: (a) $S_{C}\left(\hat{a}^{B},a^{C}\right)=0.95$; (b) $S_{C}\left(\hat{a}^{B},a^{C}\right)=0.80$. The MIIA showed accurate predictions for both binary and ternary communities as measured by cosine similarities: (a) $S_{C}\left(\hat{a}^{B},a^{B}\right)=1.00$ and $S_{C}\left(\hat{a}^{C},a^{C}\right)=0.98$; (b) $S_{C}\left(\hat{a}^{B},a^{B}\right)=1.00$ and $S_{C}\left(\hat{a}^{C},a^{C}\right)=0.94$.


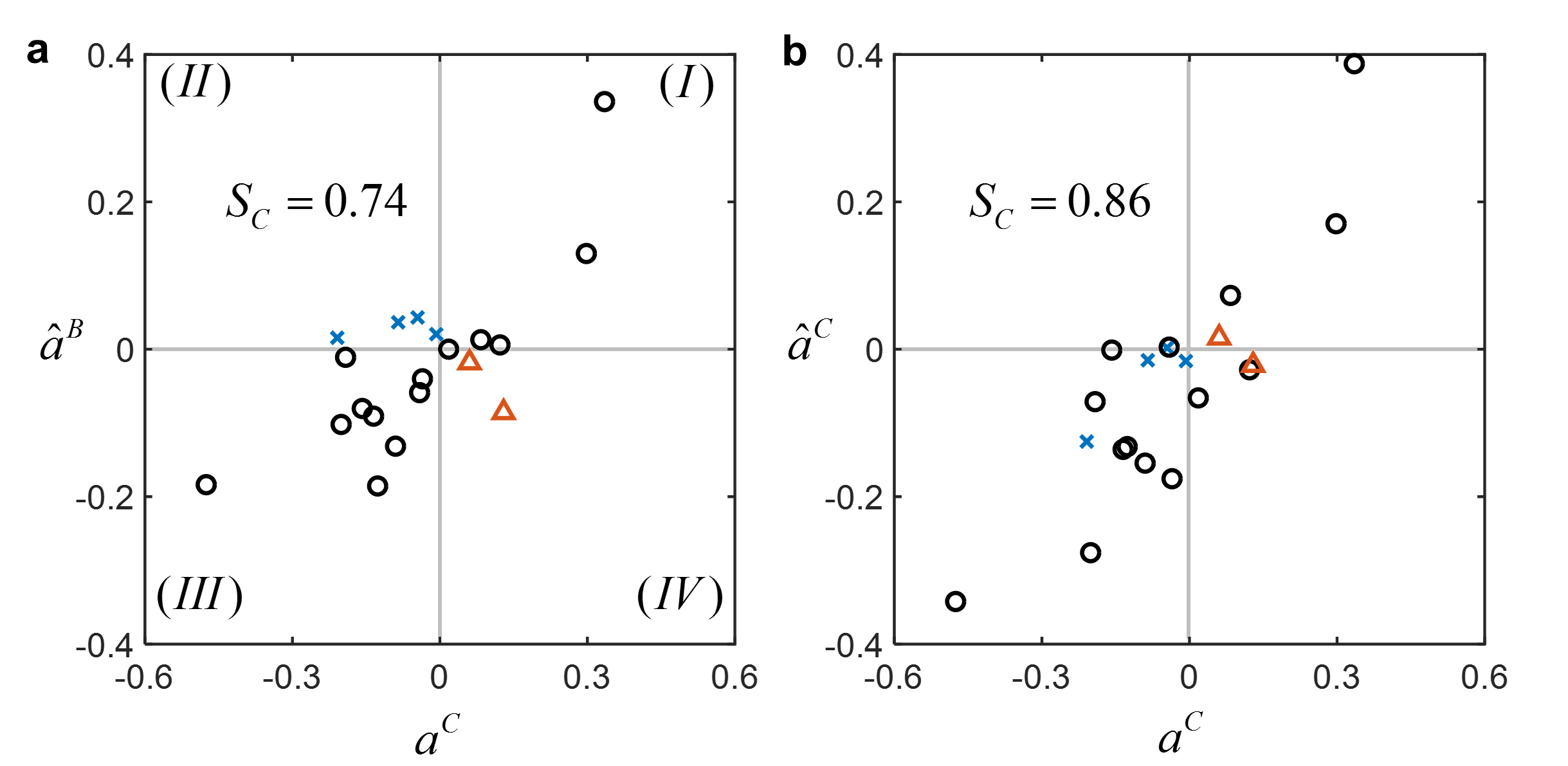


**Figure S3. Example comparison of MIIA prediction with member dependence-neglected estimation for a five member community:** (a) $\hat{a}^{B}$ vs. $a^{C}$ and (b) $\hat{a}^{C}$ vs. $a^{C}$ (where $\hat{a}^{B}$ and $\hat{a}^{C}$denote interaction coefficients predicted by neglecting and accounting for member dependence, respectively; $a^{C}$ denotes true values of interaction coefficients). $S_{C}$ is the cosine similarity (see Methods) between the interaction coefficients on x- and y-axes. As $S_{C}\left(\hat{a}^{B},a^{B}\right)$is close to 1 (results not shown), (a) also represents the relationship between $a^{B}$ and $a^{C}$ (i.e., actual modulation of interactions between binary and complex communities). Four quadrants show how the sign of interactions change between binary and complex communities. In (a), open circles on Quadrants (I) and (III) represent interaction coefficients, the sign of which were conserved from binary to complex communities. Blue crosses on Quadrant (II) and red triangles on Quadrant (IV) represent the cases where the sign of interaction coefficients were changed from plus to minus, from minus to plus, respectively. As shown in (b), MIIA provides better prediction for interactions that change direction based on the addition of new members. It needs to be noted, however, that three interaction coefficients on the fourth quadrant (i.e., two circles and one triangle) are small in their magnitudes (either actual or estimated magnitudes), implying that the sign change *per se* may not correctly evaluate the prediction of MIIA.


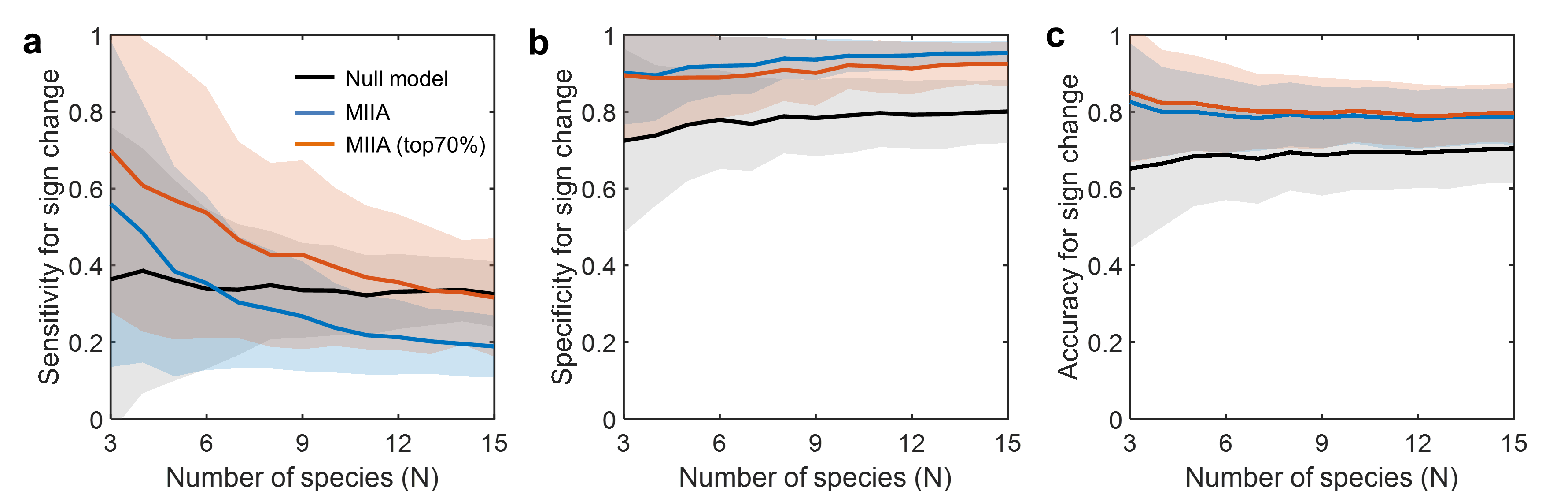


**Figure S4.** **Prediction of sign change of interaction coefficients from MIIA vs. a null model.** (a) Sensitivity for sign change, (b) specificity for sign change, and (c) accuracy for sign change, which are respectively defined $TP/\left(TP+FN\right)$, $TN/\left(TN+FP\right)$, and $\left(TP+TN\right)/\left(TP+TN+FP+FN\right)$ where TP = true positive, TN = true negative, FP = false positive, and FN = false negative. For each community of a given size (varying from 3 to 15), simulated data were sampled with the level of modulation between binary and complex communities quantified by $D_{C}\left(a^{B},a^{C}\right)$varied from 0.05 to 0.55. Lines and colored shades denote average performances and standard deviations. Black line denotes the prediction by null model, and blue and red lines are MIIA predictions for the entire set of interaction coefficients (blue) and a subset that contains only significant modulations (top 70% in magnitude or a subset of data chosen with the 30% threshold of the largest magnitude) from binary to complex communities. The null model also determines the sign change of interaction coefficients in a similar fashion. If the random number is positive (negative), the case is counted as (no) occurrence of the sign change. (a) shows that the MIIA-based predictions were not effective for predicting the occurrence of sign change as denoted by lower sensitivities because the performance was lower as compared to the null model. The performance of our method was improved when we narrowed down the predictions to the top 70% dominant predicted changes, but the resulting sensitivity was still lower than the null model except for the cases of *N* = 3 to 6. By contrast, (b) shows that the MIIA concept accurately predicted the cases where sign changes did not occur. While the performance was shown a bit lower when applied to the top 70% modulation, the specificity was significantly higher than that of the null model. In (c), the overall accuracy of predictions, in regard to sign change, was also shown higher than that of the null model, which is however due to higher specificity rather than sensitivity. That is, the MIIA provides accurate prediction for the events where the sign change did not occur (i.e., specificity), while poor prediction when the sign change occurred (i.e., sensitivity). We view that the sign change itself cannot serve as a legitimate measure for evaluating the performance due to the reason explained in Figure S3.


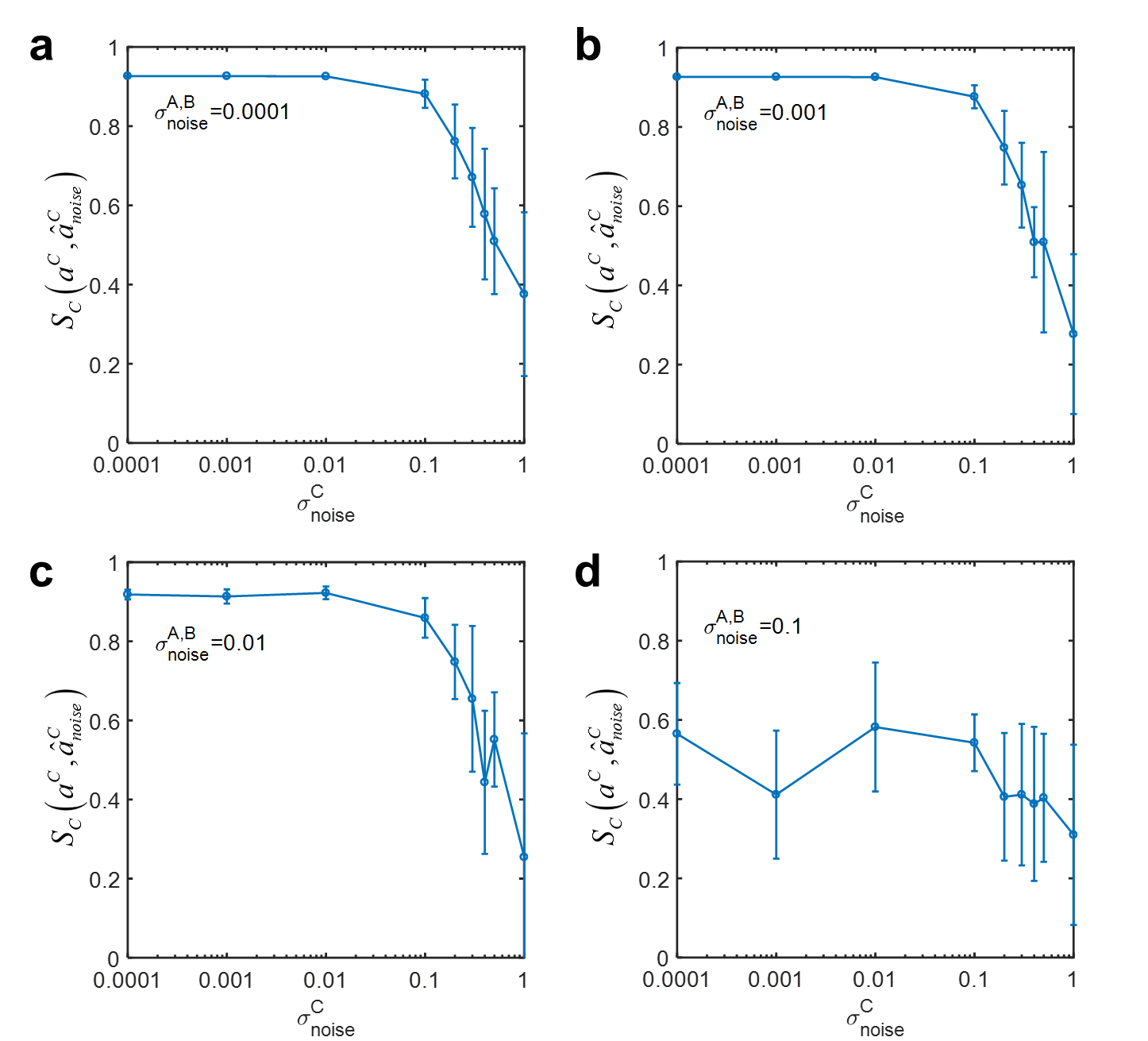


**Figure S5. Robustness of MIIA prediction against noise in complex community data for different levels of noise in axenic and binary culture data.** (a) $\delta_{noise}^{A, B}=0.0001$, (b) $\delta_{noise}^{A, B}=0.001$, (c) $\delta_{noise}^{A, B}=0.01$ and (d) $\delta_{noise}^{A, B}=0.1$


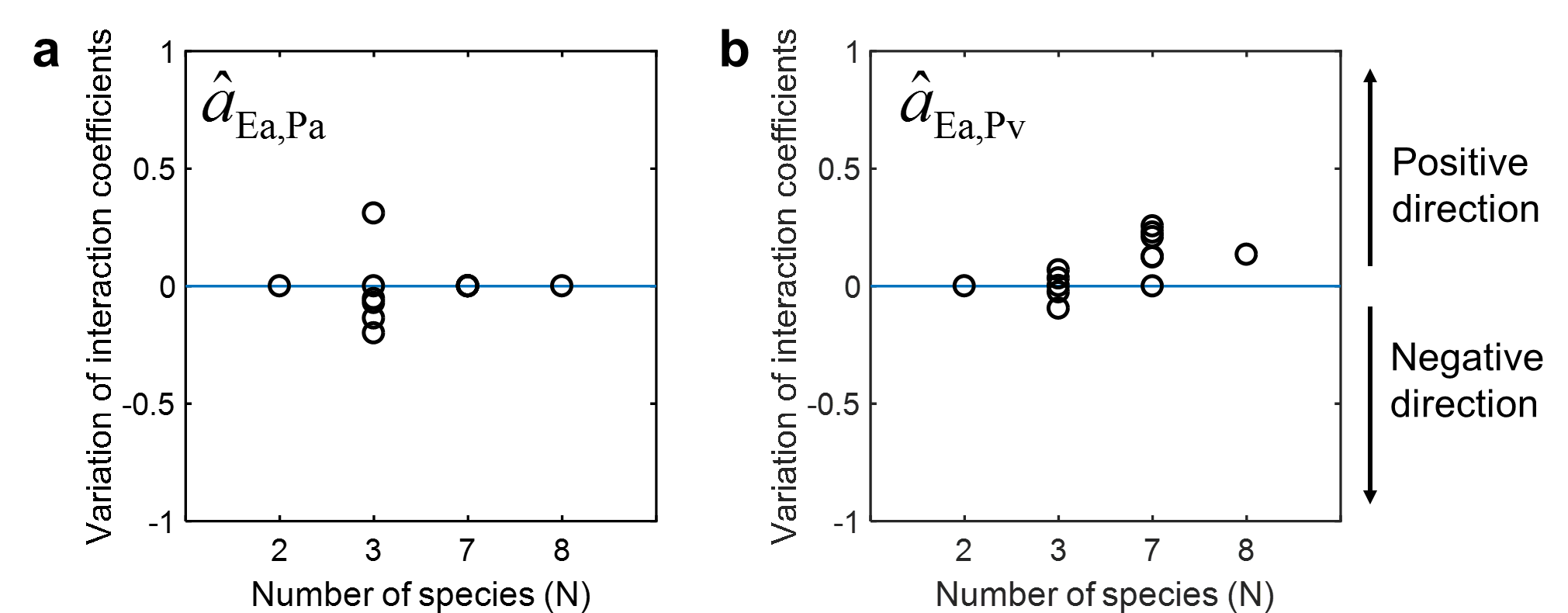


**Figure S6. Predicted modulations of interaction coefficients from binary to complex communities composed of competing bacteria considered by Friedman et al. (2017)**. (a) The effect of Pa (*Pseudomonas aurantiaca*) on the growth of Ea (*Enterobacter aerogenes*) in binary (*N*=2), ternary (*N*=3), septenary (*N*=7), and octonary cultures (*N*=8). (b) The effect of *Pv* (*Pseudomonas veronii*) on the growth of Ea for the same communities. These two figures provide zoomed-in views of two sub-graphs in Figure 5(a) (i.e., the first two sub-graphs from the left on the top row). See the caption of Figure 5 for all other details.


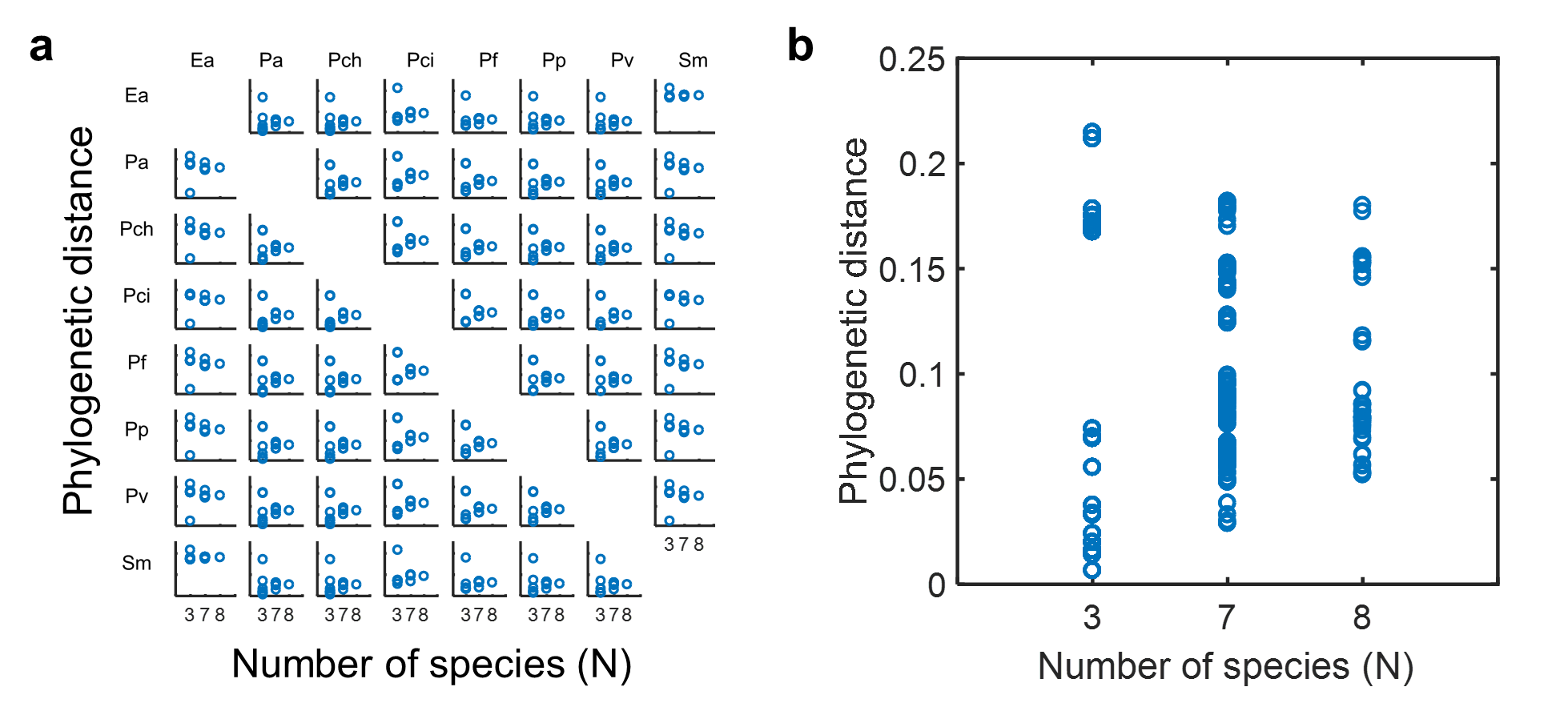


**Figure S7. Phylogenetic distances between effectors and added species.** (a) The complete array of phylogenetic distances between the effector species (i.e., each species in column) and added species. (b) Overall trend obtained by merging all individual plots in (a) into one place. As in Figure 5, the species in columns and rows in (a) represent the affecting species (effector) and the affected species, respectively. Open circles in each sub-graph represent phylogenetic distances between the effector and additionally added species. Thus, this gives an indirect measure of functionally similarity between them. The scales of x- and y-axis are the same in all cases of (a) and (b): y-axis varies from 0 to 0.25; on the x-axis, the numbers 3, 7 and 8 denote ternary, septenary, and octonary cultures, respectively. Phylogenetic distances were averaged out among added members, when more than one species was added.
